# Contaminant Removal Using Vibrating Surfaces: Nanoscale Insights and a Universal Scaling Law

**DOI:** 10.1021/acs.nanolett.4c05973

**Published:** 2025-03-05

**Authors:** Rohit Pillai, David Neilan, Cameron Handel, Saikat Datta

**Affiliations:** Institute for Multiscale Thermofluids, School of Engineering, University of Edinburgh, Edinburgh EH9 3FD, United Kingdom

**Keywords:** molecular dynamics, surface acoustic waves, nanoparticle removal, self-cleaning surfaces

## Abstract

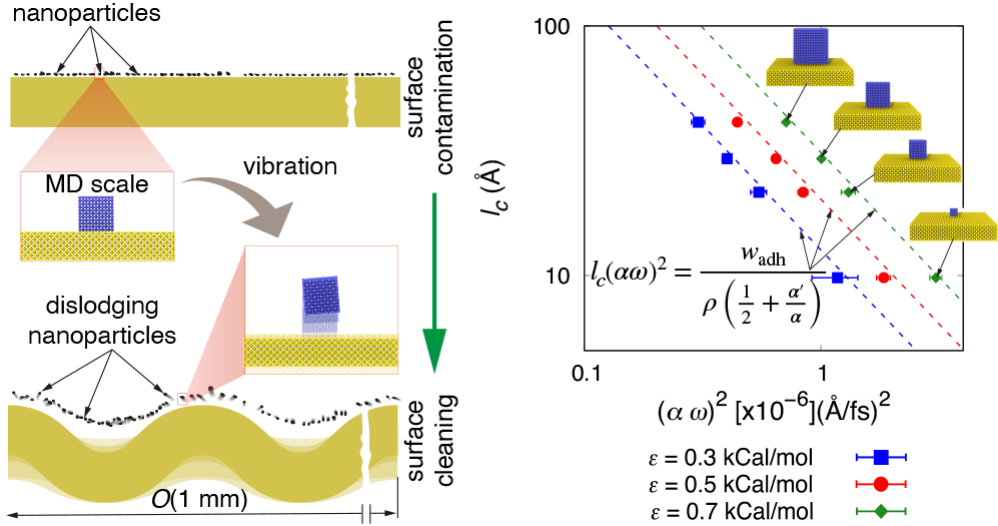

The development of active self-cleaning surfaces, i.e., surfaces that remove nanoscale contaminants using external forces such as electric or magnetic fields, is critical to many engineering applications. The use of surface vibrations represents a promising alternative, but the underlying nanoscale physics, in the absence of an intermediate liquid medium, is poorly understood. We used molecular dynamics simulations to explore the use of ultra-high-frequency surface acoustic wave devices for contaminant removal. Our simulations reveal that there exists a critical vibrational energy threshold, determined by the amplitude and frequency of the surface vibrations, that must be surpassed to effectively dislodge contaminant particles. We derive a universal scaling law that links the characteristic size of particles to the optimal vibrational parameters required for their removal. This provides a theoretical framework to aid the development of advanced, scalable self-cleaning surfaces with applications ranging from semiconductors to large-scale industrial systems.

The removal of contaminants from surfaces is widespread both in nature, as seen in the self-cleaning properties of lotus leaves^1^ and organisms such as cicadas and geckos,^2−5^ and in engineering applications, including pharmaceutical manufacturing^6^ and food processing.^7^ Contaminant removal in both natural systems and industrial applications can be categorized into passive methods,^1,8,9^ which do not require external energy input, and active methods,^10−17^ which involve the application of energy through mechanical, thermal, electrical, or chemical means. A variety of active methods, such as ultrasonic^11^ and megasonic cleaning,^12,13,17^ high-speed air jet removal,^14^ droplet sprays,^15^ and cryo-aerosols,^16^ are routinely employed in industry. Regardless of the technique used, contaminant removal in active methods is achieved when sufficient inertia is imparted to the particle, so that it overcomes adhesive forces and detaches from the surface. This inertia can be expressed as *ma*, where *m* represents the mass of the particle and *a* is the acceleration imparted by an external stimulus. For micrometer- to submicrometer-sized particles, their relatively larger mass (*m*) means that a lower acceleration (*a*) is sufficient to generate enough inertia to overcome adhesive forces. However, recent engineering applications, such as semiconductor fabrication and cleaning,^17−20^ increasingly require the effective removal of nanoscale-sized contaminants. Due to the decreasing mass of these smaller particles, higher accelerations are needed to produce sufficient inertial forces for particle removal, which is challenging for current methods.

Surface acoustic waves (SAWs) represent one of the few methods that can generate inertia in micro- and nanoscale applications, because of the ultrahigh frequencies that can be generated (up to ∼34 GHz^21^). SAW devices are well-established and offer several advantages, such as durability, cost-effectiveness, and ease of mass production.^22,23^ Previous studies have used vibrating surfaces to prevent polymer deposition,^24^ move droplets,^25,26^ or drive acoustic streaming within liquids^27^ for cleaning purposes, mitigate dust in space environments,^28^ remove contaminants from silicon wafers,^29^ and remove fouling materials from solar panel surfaces.^30^ Note that SAWs can be used to remove contaminants by either vibrating the surface under the contaminant directly or driving acoustic waves through an intervening liquid. In the case of the former, the applications typically involved removal of larger particles.^24,30^ In the latter case, acoustic cavitation and streaming within the liquid gain importance.^25−27^ In both cases, much lower frequencies are used relative to what is possible today,^21^ and the contaminants are much larger than those found in applications like semiconductor cleaning.^17^ Thus, no literature on the high-frequency SAW removal of nanoscale contaminants exists.

We therefore propose the use of high-frequency SAWs as a novel active method to remove nanoscale contaminants (or nanoparticles) from surfaces. We envisage the integration of piezoelectric materials to produce self-cleaning surfaces that generate vibrations at desired frequencies (schematic in Figure 1). We perform molecular simulations to provide a proof of concept and then go on to elucidate the underlying mechanisms that govern this process. We then developed a generalized theoretical framework that can bridge the gap between nanoscale simulations and practical engineering applications, paving the way for the design of advanced active self-cleaning surfaces that operate efficiently across different scales.

**Figure 1:**
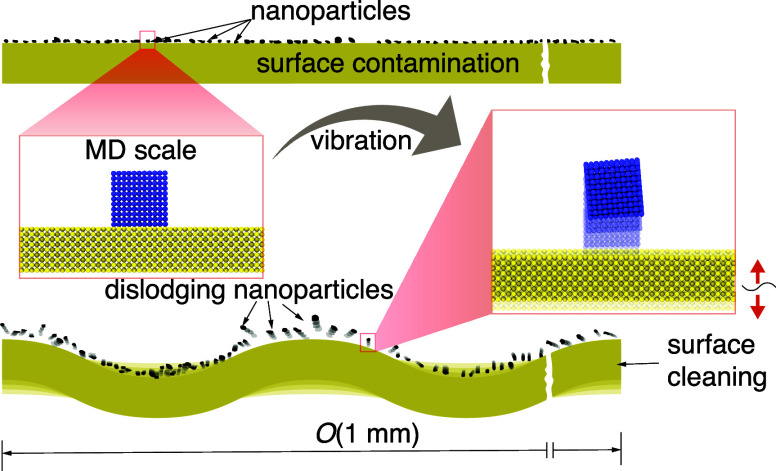
Schematic of the proposed method for dislodging nanoparticles using SAWs. The insets provide close-ups of individual nanoparticles (simulation domain used in this study), showing nanoparticles placed on a surface that undergoes vertical vibrations.

We employ nonequilibrium molecular dynamics (NEMD) simulations in this study. The NEMD setup consists of a nanoparticle (representing a nanoscale contaminant) positioned on a solid surface (see the insets of Figure 1). The wavelengths in SAW devices used in microfluidic applications range from approximately micrometers to millimeters. In the current study, the NEMD setup represents a small spanwise segment of such a device; as a result, the width of the domain is significantly smaller than the wavelengths employed. Consequently, the motion generated by a SAW can be approximated as a vertical oscillatory motion, described by the equation *z* = *z*_0_ + α sin(2*πft*), where α is the amplitude and *f* is the frequency, applied to the solid surface about its equilibrium position *z*_0_. A detailed description of the simulation methodology is provided in the Supporting Information. We aim to investigate particle detachment using only the initial oscillation of vibration, as would be created by a SAW impulse.^31−33^ The adhesion forces between the nanoparticle and the surface are modeled by van der Waals (vdW) interactions in this work. However, note that we go on to show that the mechanism of SAW-mediated particle removal is governed by the total energy of adhesion (i.e., the work of adhesion), regardless of the specific nature of the forces^34^ or contact mechanics involved.^35−39^ Since we focus on the energy of adhesion, which is a thermodynamic property, our analysis is universal, meaning it applies to any adhesion scenario, irrespective of the forces responsible for the adhesion. Extending our results to incorporate long-range electrostatic interactions between a surface and a particle, for example, is fairly straightforward, as shown in the Supporting Information.

To assess the viability of SAW-driven nanoparticle dislodging, we varied the time period of vibration *T* (for constant amplitude α) for different interaction strengths ε between the nanoparticle and the surface. We identified three possible outcomes across our parametric space (see Figure 2) when the surface is vibrated: (1) clear lift-off, where the supplied vibrational energy comfortably overcomes the adhesive forces (Figure 2a); (2) optimum lift-off, where the vibrational energy just suffices to overcome the adhesive forces (Figure 2b); and (3) non-lift-off, where the vibrational energy is insufficient to overcome the adhesive forces (Figure 2c). The criteria used to identify optimum lift-off are provided in the Supporting Information.

**Figure 2:**
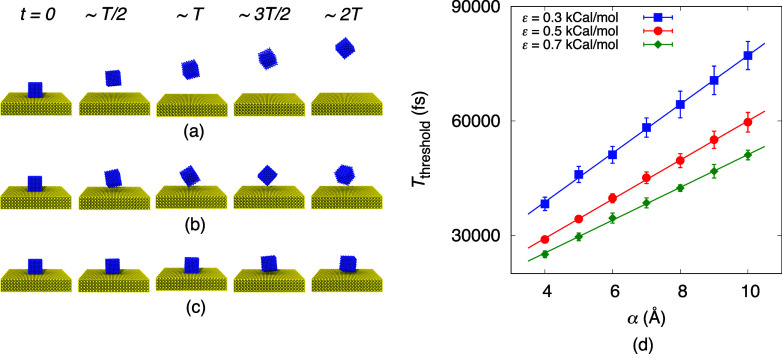
Illustrations of (a) clear lift-off for ε = 0.7 kcal/mol, α = 4 Å, and *T* = 21 739.1304 fs (*f* = 46 GHz), where the supplied energy significantly exceeds the requirement, (b) optimum lift-off for ε = 0.7 kcal/mol, α = 4 Å, and *T* = 26 315.78947 fs (*f* = 38 GHz), when the supplied energy approximately equals the work of adhesion, and (c) non-lift-off for ε = 0.7 kcal/mol, α = 4 Å, and *T* = 28 571.4286 fs (*f* = 35 GHz), when the supplied energy falls short of overcoming the work of adhesion. (d) Variation of the time period for optimum lift-off (*T*_threshold_) at different vibration amplitudes (α). The results are based on five realizations, with error bars representing the 95% confidence interval. The lines represent linear fits across the data points.

We define the time period at which optimum lift-off occurs as the threshold time period (*T*_threshold_), and we plot *T*_threshold_ as a function of vibration amplitude (α) in Figure 2d. We observe that (i) *T*_threshold_ increases linearly with α in all cases and (ii) the actual magnitudes of *T*_threshold_, as well as the slope between *T*_threshold_ and α, decrease as ε increases. This implies that the optimum lift-off of a particle depends on the circular velocity (*αω*, where *ω* = 2π/*T*) of the vibrating surface and ε (i.e., interaction strength) between two materials.

As the nanoparticle detaches due to the transfer of energy from the mechanical work performed by the moving surface, we track both the force between the nanoparticle and the surface and the work performed during nanoparticle detachment. Figure 3a illustrates the temporal variation of force on the nanoparticle during lift-off. Snapshots of the molecular trajectory (from a representative simulation), magnifying the nanoparticle–surface interface, are displayed in the central panel and correspond to the points indicated by the markers. Here, a positive force represents repulsion while a negative force indicates attraction. As shown in Figure 3a, the initial force between the nanoparticle and the surface is ∼0 at equilibrium. However, as the surface moves toward the nanoparticle, the relative displacement decreases (i.e., *d*_i_ > *d*_ii_ > *d*_iii_ > *d*_iv_), leading to an increase in the repulsive force as molecules overlap, as observed through stages i–iv. After reaching a maximum value, the repulsive force begins to decrease as the nanoparticle moves away from the surface through stages v–vii (i.e., *d*_v_ < *d*_vi_ < *d*_vii_). At a point near stage vii, the force between the nanoparticle and the substrate becomes zero (at time *t*_*F*=0_). Beyond this point, the attractive vdW force governs the subsequent nanoparticle dynamics. The magnitude of the attractive force initially increases with relative displacement, reaching a minimum near stage viii. As the nanoparticle moves farther from the surface, the attractive vdW force gradually weakens and eventually diminishes to zero beyond the cutoff distance.

**Figure 3:**
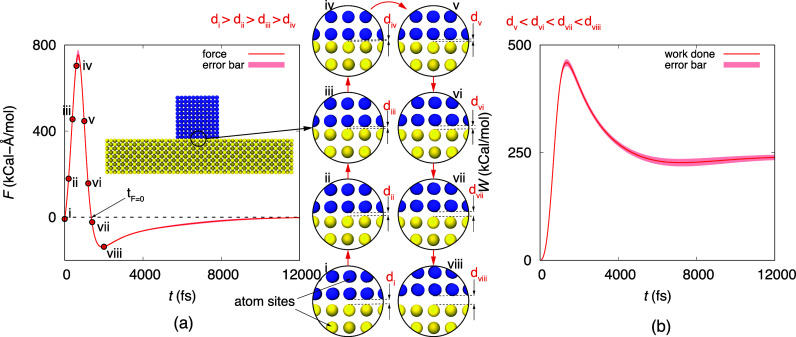
(a) Variation of the force between the nanoparticle and the surface with time during the optimum lift-off case for α = 4 Å and ε = 0.5 kcal/mol. The values shown are averaged from five individual realizations. The central panel displays snapshots of the molecular trajectory magnifying the nanoparticle–surface interface at the points indicated by the markers on the force–time curve. The atoms displayed in snapshots are actual atom positions from our molecular simulations and are therefore in scale (relative to each other), enabling comparison of the distances (*d*) between the particle and surface atoms shown in all cases. (b) Temporal variation of the cumulative work done against the interatomic forces during nanoparticle detachment.

The work done by the surface at a time step *t* can be calculated as
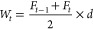
1where *F*_*t*–1_ is the force between the particle and the substrate at time (*t* – 1), *F*_*t*_ is the same but at time *t*, and *d* is the displacement of the particle relative to the surface (between times *t* – 1 and *t*). Cumulative work *W* is obtained by adding *W*_*t*_ for successive time steps. Note that eq 1 considers *W*_*t*_ to be positive and negative when the relative displacement is toward and away from the direction of the force, respectively. Figure 3b shows the cumulative work done (*W*) by the surface on the particle as a function of time *t* for the optimum lift-off case shown in Figure 3a. In the initial stages of optimal lift-off, the relative displacement is opposite to the repulsive force, as the surface moves toward the particle (see center panels i–iv in Figure 3). This results in an extremely steep slope for *W* in Figure 3b. As the particle starts moving away from the substrate (stages v and vi), the work–time curve quickly changes in slope and reaches a maximum (at *t*_*F*=0_). Beyond this point, the attractive force comes into play and the relative displacement occurs in the direction of the force. Due to the negative work done, *W* starts decreasing in the plot until it reaches a steady value. Once the particle reaches the cutoff, there is no longer any interaction between the particle and the substrate, and *W* stays unchanged.

Analyzing work–time plots for several of our cases, we hypothesize that the qualitative variation in *W* (over the lifetime of an optimal lift-off process) is universal, as it is not a vibration–parameter specific calculation. In other words, *regardless of the frequency and amplitude of the surface vibration or the strength of particle–surface interactions, the optimum lift-off process proceeds in exactly the same way*. We observed that the change in potential energy between times zero and *t*_*F*=0_ is negligible in all of our cases. Consequently, the cumulative work done, *W*, until *t* = *t*_*F*=0_ is retained as kinetic energy in the nanoparticle at *t* = *t*_*F*=0_. At the initiation of vibration, the relative velocity between the nanoparticle and the surface is −*αω*, while subsequently, the relative velocity shifts to *αω* at *t* = *t*_*F*=0_. Therefore, the nanoparticle must acquire kinetic energy equivalent to the energy required for this change in velocity, i.e., KE = (1/2)*m*(2*αω*)^2^. This is shown quantitatively in Figure 4a–c, where the calculated kinetic energy, based on *α* and *ω*, is compared with cumulative work *W*, demonstrating near-perfect agreement. Given that the kinetic energy depends on both *α* and *ω* and that energy conversion occurs similarly up until *t* = *t*_*F*=0_, it can be asserted that the phenomenon exhibits a dynamic similarity. To verify this, we plotted the variation of normalized relative velocity V* (V* = *v*/*αω*) at the initial stages of detachments as a function of normalized time t* (t* = *t*/*t*_*F*=0_). Negative and positive values of V* signify that the nanoparticle and the surface are moving toward and away from one another, respectively. We then analyzed the change of the normalized center of mass distance, *D*_CM_^*^ (*D*_CM_/(*αω* × *t*_*F*=0_), where *D*_CM_ is the change of center of mass distance from the initiation of vibration), between the nanoparticle and surface with t*, shown in Figure 4g–i. The agreement in the results confirms that acoustic parameters *α* and *f*, along with interaction strength ε, are interdependent. This enables us to develop a new theoretical framework to characterize this process, as discussed below.

**Figure 4:**
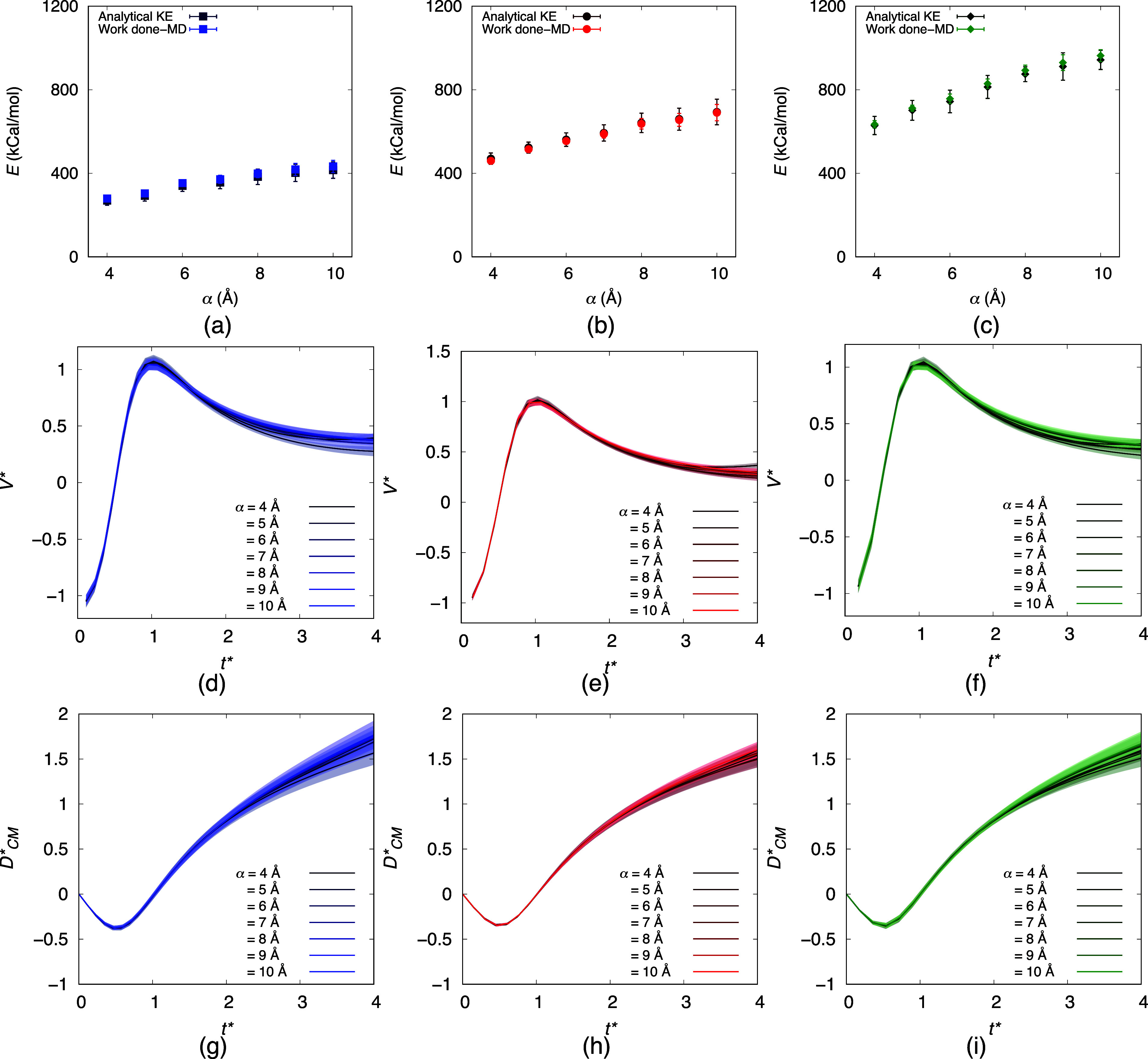
(a–c) Comparison of the kinetic energy of the nanoparticle estimated from the optimum α and ω (analytical KE) and obtained from MD employing eq 1. (d–f) Variation of the normalized center of mass velocity of the nanoparticle with normalized time at the initial stages of detachments. (g–i) change of the normalized center of mass distance between the nanoparticle and surface with normalized time. The blue, red, and green shades represent ε values of 0.3, 0.5, and 0.7 kcal/mol, respectively.

## Universal Scaling Law

We track the energy variation of the nanoparticle during lift-off for all cases to formulate an energy balance. Note that in all cases of optimal lift-off, the energy interactions between the surface and the particle occur when the interface of the particle remains close to the surface, causing the detachment to be ensured at the end of the upward motion. If this detachment threshold is met before the end of the upward motion, the particle retains high kinetic energy and experiences a clear lift-off instead, as shown in Figure 2a. In the case of optimal lift-off, the total energy gained due to the work done by the moving surface up until *t*_*F*=0_ (shown in Figure 3), , is utilized to perform displacement work beyond *t*_*F*=0_, , as the particle moves away from the surface and to overcome the work of adhesion, *W*_adh_. The particle also retains a minimum amount of kinetic energy (KE) at the end of the upward movement of the surface. Therefore, the energy balance about *t*_*F*=0_ (i.e., energy gain up to *t*_*F*=0_ = energy expenditure after *t*_*F*=0_) can be expressed as follows:
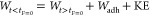
2

**Figure 5:**
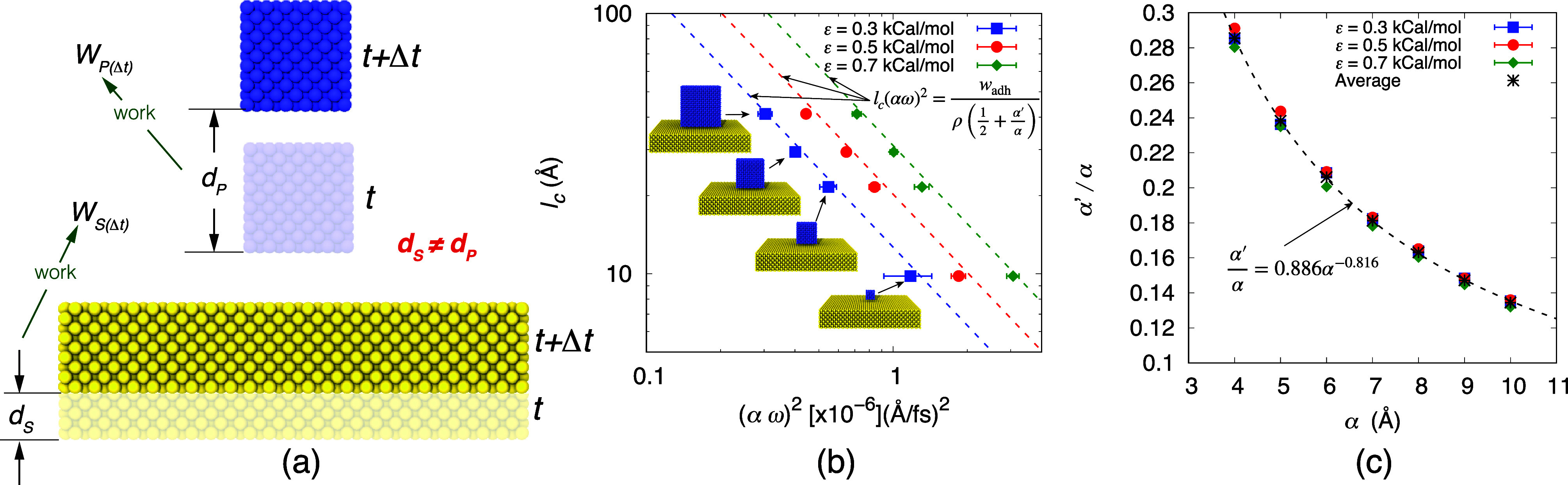
(a) Schematic illustration of the displacement of the nanoparticle and the surface over a time interval Δ*t*. (b) Comparison of the derived scaling law with the results obtained from MD simulations. (c) Variation of α′/α for optimum lift-off of the nanoparticle as a function of amplitude α. The black dotted line represents the fitted curve over the averaged α′/α from all interaction strengths.

As panels a–c of Figure 4 make evident, the work done up to point *t*_*F*=0_ is equal to the kinetic energy of the particle, i.e., .  arises due to the change in the distance between the particle and the surface before detachment and is caused by the difference in velocity between the upward motion of the nanoparticle and the surface beyond *t*_*F*=0_. Figure 5a illustrates the displacement of the particle (*d*_P_) and the surface (*d*_S_) over a time interval Δ*t*, resulting in a change in distance between the nanoparticle and the surface (Δ*d* = *d*_P_ – *d*_S_). Therefore, we can define , where *W*_P_ is the work caused by the displacement of the particle, corresponding to *d*_P_, and *W*_S_ is the work resulting from the surface displacement, associated with *d*_S_. For mathematical convenience, *W*_P_ is estimated by considering the moving surface as the frame of reference while *W*_S_ is estimated by assuming the particle as the reference frame. Starting with *W*_P_, as the surface is considered fixed, the work done results from the particle moving away from the surface (against the vdW attraction) and is equal to the change in the kinetic energy of the particle. Thus, given that the relative velocity between the particle and the surface decreases from *αω* initially to zero when the interaction strength between them becomes negligible, *W*_P_ can be expressed as
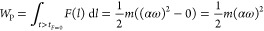
3where *F*(*l*) is the force on the particle at a distance *l* from the surface and *m* is the mass of the particle.

For *W*_S_, as the surface undergoes sinusoidal vibrational motion while the particle is assumed to be fixed, it decelerates with a magnitude of |*ω*^2^*y*| at a displacement *y*. The magnitude of the force on the particle at *y* due to the motion of the surface is |*mω*^2^*y*| (i.e., the product of mass and deceleration). Considering the displacement of the surface at *t*_*F*=0_ to be α′, the total work done due to the surface motion beyond *t*_*F*=0_ can be expressed as
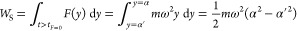
4

Next, the kinetic energy of the particle at the end of the upward motion of the surface in eq 2, KE, emerges due to the change in the deceleration of the surface during its upward motion. The excess energy, arising from the change in the deceleration, is retained by the particle after the surface stops at the topmost position. The kinetic energy resulting from this change in deceleration can be simply obtained from the equation *v*_2_^2^ = *v*_1_^2^ + 2*as* (where *v*_2_ and *v*_1_ are the final and initial velocities, respectively, and *a* and *s* are the acceleration and displacement), such that
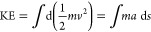
5

As the particle moves along with the surface, its displacement beyond *t*_*F*=0_ is the same as that of the surface, α – α′, and it undergoes a similar change in deceleration with displacement *s* as the surface, i.e., ω^2^*s*. Therefore
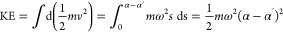
6Substituting the expressions for work done and kinetic energy from eqs 3, 4, and 6 into eq 2 results in
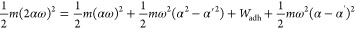
7

Finally, we can express *W*_adh_ and *m* as *W*_adh_ = *Aw*_adh_ and *m* = *ρV*, where *A* and *w*_adh_ are the cross-sectional area of the particle and the work of adhesion per unit area, respectively; and *ρ* and *V* are the density and volume of the particle, respectively. Rearranging eq 7, we develop our scaling law:
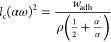
8where *l*_c_ is the characteristic length obtained by dividing the volume by the cross-sectional area (*l*_c_ = *V*/*A*). Note that eq 8 is valid for short-range interactions, such as van der Waals forces. However, for interactions that change more gradually, such as electrostatic forces, the detachment threshold is not met at the top of the upward motion of the surface. Instead, the surface continues to do work while moving downward. This changes the denominator of the scaling equation from  to , while keeping the form the same. A detailed derivation of the scaling with the presence of electrostatic interaction is provided in the Supporting Information.

α′/α in eq 8 represents the normalized displacement of the surface in its oscillatory motion during which the nanoparticle accumulates the energy necessary for the detachment, which is independent of domain size and can be determined a priori. Figure 5b presents a comparison between eq 8 and the results from MD simulations, illustrating the variation of *l*_c_ as a function of (*αω*)^2^ for optimal lift-off cases on a log–log scale for different interaction strengths. We test the validity of our proposed scaling relation by considering four different particle sizes, namely, 9.8, 21.56, 29.4, and 41.16 Å, which are approximately 0.5, 1, 1.5, and 2 times the primary particle size used in the study, respectively. The optimal α′/α used in eq 8 for plotting the dotted lines is taken from the cases with a particle size of 21.56 Å. Figure 5c shows that while the normalized displacement, α′/α, varies with α, it does not depend on interaction strength ε or, therefore, on *w*_adh_. This indicates that it is characteristic of the amplitude of the vibration, as verified in Figure 5c, where α′/α values are provided for the vibrational amplitudes typical of high-frequency SAW devices. Note that *w*_adh_ in eq 8 is a thermodynamic quantity and does not depend on the nature of the forces giving rise to it, be it van der Waals, electrostatic, or capillary forces. Therefore, the scaling law obtained in this study is universal and applies to any material–species combination in SAW-driven particle removal. If the work of adhesion for a given material–species pair is known, eq 8, along with the fitted curve in Figure 5c, can be used to design vibration-driven nanoparticle manipulation devices for a wide range of scenarios, well beyond what is feasible to study by using molecular simulations.

In conclusion, this study proposes a novel method for nanoparticle removal by using SAWs. Through molecular dynamics (MD) simulations, we explore the interplay between vibrational parameters and the forces that govern the adhesion and detachment of nanoparticles from surfaces. We demonstrate the existence of a critical threshold in vibrational energy required to overcome the adhesive forces between the nanoparticles and surfaces. The energy transfer during the lift-off process is governed by a balance between the work done by the surface to overcome adhesion and the kinetic energy retained by the particles post detachment. This energy balance enables us to derive a universal scaling law that relates the characteristic lengths of particles to the vibrational parameters required for their removal. This scaling law, validated by our MD simulations, provides a predictive tool for nanoparticle removal for any vibrating surface as long as the interaction strength between them can be quantified. This scaling law can guide the development of SAW-driven nanoparticle manipulation devices. The findings have significant implications for a range of applications, from maintaining microelectronic devices to developing scalable technologies for industrial surface cleaning. Future work could focus on exploring the effects of surface roughness and material heterogeneity on nanoparticle removal.

## Data Availability

The data and the script files for selected cases that support the findings of this study are available at https://datashare.ed.ac.uk/handle/10283/8949.

## Supporting Information Available

Details of the simulation methodology, optimum detachment criteria, work of adhesion calculation, effect of drag, and extending the framework to include electrostatic effects (PDF)
