# Supplementary material for: Contaminant Removal Using Vibrating Surfaces: Nanoscale Insights and a Universal Scaling Law
Source: Nano Lett. 2025 Mar 5;25(11):4284–90. doi: 10.1021/acs.nanolett.4c05973 (PMC11926963; doi:10.1021/acs.nanolett.4c05973)
Supplement: Supplementary file 1 — nl4c05973_si_001.pdf [file nl4c05973_si_001.pdf]

Supporting Information: Contaminant removal using vibrating surfaces: nanoscale insights and a universal scaling law

Rohit Pillai,* David Neilan, Cameron Handel, and Saikat Datta*

Institute for Multiscale Thermofluids, University of Edinburgh, Edinburgh EH9 3FD, United Kingdom

E-mail: r.pillai@ed.ac.uk; saikat.mech@gmail.com

This document includes detailed information on the methodology and simulation setup for the molecular dynamics simulations, the criteria for determining the optimal detachment cases, the details of the adhesion work calculations, and an investigation into the influence of drag and electrostatics on the scaling law proposed in the Letter.

Simulation Methodology

Non-equilibrium molecular dynamics simulations are performed to analyze vibration-driven nanoparticle detachment, where the motion of each particle is determined by solving Newton's equation of motion. For a particle of mass m_i, the equation can be written as:

$$F_i = \sum_{i \neq j}^{N} -\frac{\mathrm{d}U_{ij}}{\mathrm{d}r} \frac{r_{ij}}{|r_{ij}|} = m_i a_i = m_i \frac{\mathrm{d}^2 r_i}{\mathrm{d}t}; \tag{1}$$

where a_i and r_i are the acceleration and position vectors of atom i, respectively. F_i represents the force on the atom, which is obtained from the interaction potential U_{ij} between atom i

and any atom j in the system, modelled by the 12-6 Lennard-Jones potential:

$$U_{ij} = 4\varepsilon_{ij}\left[\left(\frac{\sigma_{ij}}{r_{ij}}\right)^{12} - \left(\frac{\sigma_{ij}}{r_{ij}}\right)^{6}\right]; \tag{2}$$

where r_{ij} is the distance between atoms i and j. ε_{ij} and σ_{ij} are the depth of the potential well and the effective diameter (the distance at which the inter-atomic potential is zero) of the atoms. N is the total number of atoms in the system. To enhance computational efficiency, the Lennard-Jones (LJ) interactions between molecules are truncated radially at 15 Å. Both the nanoparticle and the surface are composed of an FCC crystal structure with a lattice constant of 3.92 Å . The σ and ε for nanoparticle-nanoparticle and surface-surface interactions are set to 2.471 Å and 15.9743 kCal/mol, respectively. The atomic mass is 195.084 g/mol for atoms in both the nanoparticle and the surface. The interaction strength between the nanoparticle and the surface is varied at $\varepsilon = 0.3$, 0.5, and 0.7 kCal/mol to analyse the effect of different surface energies (work of adhesion) on nanoparticle detachment.

Figure S1 illustrates the initial configuration of the simulation domain. The surface is constructed by stacking 9 layers of the FCC crystal, with a cross-sectional area of 94.08 $\times$ 94.08 Å^2. The domain boundaries are periodic in all directions. The domain height is set to 220 Å to allow the nanoparticle to move far enough away from the surface without interacting with it through the periodic boundaries along the vertical direction. For simplicity, the nanoparticle is shaped like a cube. In most simulations, a cubic nanoparticle measuring 21.56 Å and consisting of 666 atoms is used for detachment studies. However, for the scaling analysis, the nanoparticle size is varied to 9.8 Å (63 atoms), 21.56 Å (666 atoms), 29.4 Å(1688 atoms), and 41.16 Å (4631 atoms).

All simulations are carried out using the open-source MD software LAMMPS.[1] To analyze the dynamics of the nanoparticle interacting with the vibrating surface while minimizing noise from thermal fluctuations, the nanoparticle is modeled as a rigid body. Consequently, at each time step, the total force and torque on the nanoparticle are calculated as the sum of the forces and torques on its constituent particles. The coordinates and velocities of the

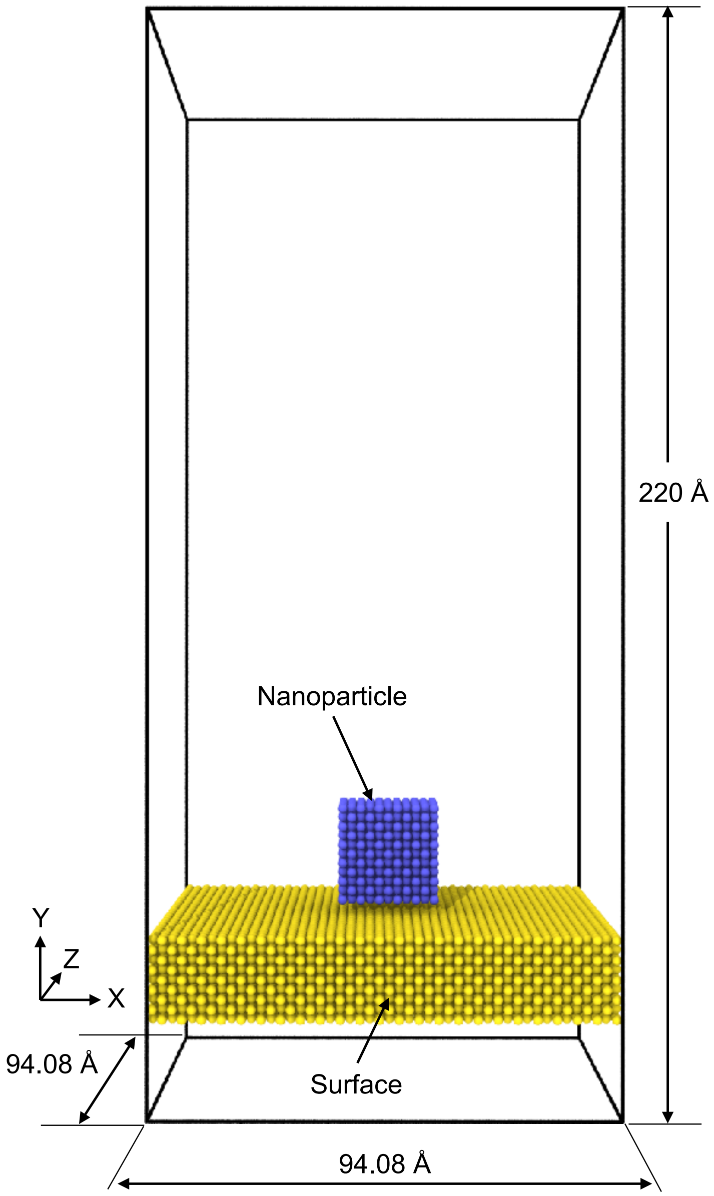

Figure S1: Initial configuration of the domain used in nanoparticle detachment simulations

atoms in the nanoparticle are updated to ensure it moves and rotates as a single entity. For the vibrational motion, the surface is also time-integrated to move as a rigid body in a sinusoidal path.

The particle in the initial configuration shown in Figure S1 is integrated into a canonical (NVT) ensemble for 10 ns to stabilize it on the surface at equilibrium. The nanoparticle is allowed to exchange energy with a Nose–Hoover[2–4] thermostat maintained at a temperature

of 300 K. Time integration during the equilibration is performed using the time-reversible and symplectic integration scheme developed by Kamberaj et al.,[5] with a time step of 1 fs. Since the nanoparticle is modeled as a single rigid body, estimating its temperature would yield results with large fluctuations and noise. Therefore, the interaction energy between the surface and the nanoparticle is tracked instead of the particle temperature to monitor whether the system has reached an equilibrium state. Figure S2 shows the evolution of the surface-nanoparticle interaction energy over time during equilibration, which does not exhibit significant change after a large variation in the initial stages.

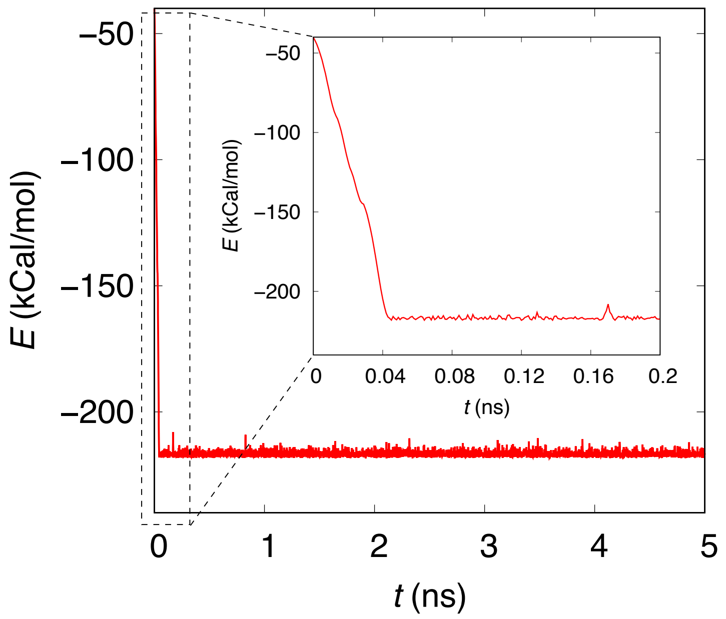

Figure S2: Variation of the surface-nanoparticle interaction energy as a function of time during equilibration (first 5 ns for $\varepsilon = 0.5$ kCal/mol). The inset highlights the significant initial change.

Five realizations of each vibrational parameter are performed to estimate the uncertainty associated with MD simulations of the nanoparticle dislodging. Five restart files are generated 2 ns apart after equilibration mentioned above for the realizations, ensuring that the final atomic trajectories at restart files are not correlated. In each realization, the nanoparticle is further equilibrated for 1 ns using the aforementioned NVT scheme with 0.1 fs time steps. Vibration is then imparted to the surface, and the position and velocity of the atoms

in the nanoparticle are updated through NVE time integration with a 2 fs time step using the symplectic quaternion scheme developed by Miller et al.[6]

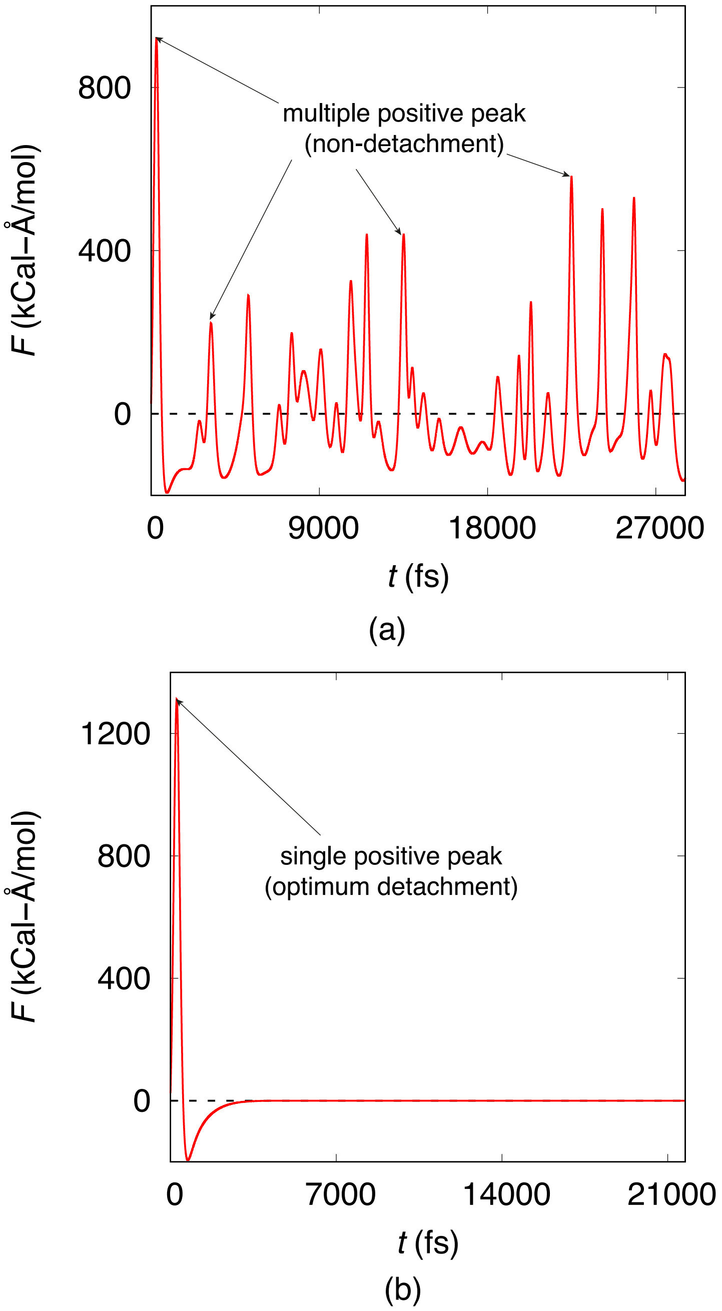

Figure S3: Variation of the force between the nanoparticle and the surface in a (a) non-detachment and an (b) optimum detachment case.

Optimum Detachment Criteria

The force between the nanoparticle and the surface is monitored to identify detachment. At equilibrium, this force is approximately zero, but during surface vibration when the distance between the surface and nanoparticle is less than the equilibrium distance, the force becomes repulsive. Conversely, the force becomes attractive when the relative distance exceeds the equilibrium position. Since energy is conserved, the work done by the surface against the repulsive force is stored as the kinetic energy of the nanoparticle. If the kinetic energy is sufficient to overcome the work of adhesion, detachment occurs. Figure S3 depicts the force between the nanoparticle and the surface for both a detachment and non-detachment case. Multiple positive peaks in figure S3(a) indicate that the particle contacts the surface more than once during the vibration. Therefore, for a given amplitude, the minimum frequency at which there is only one positive peak in the force vs. time plot during the first two surface oscillations, as shown in Figure S3(b), is considered the optimum vibrational parameter for detachment.

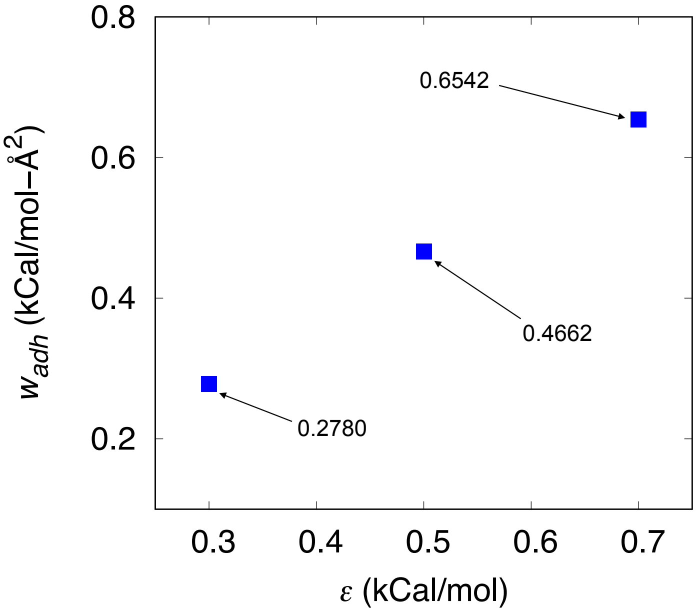

Figure S4: Variation of work of adhesion per unit area (w_{adh}) with surface/nanoparticle interaction strength (ε).

Work of Adhesion

The work of adhesion is calculated using equation 2 between the atoms of the nanoparticle and the surface during the second equilibration step mentioned above. The data is averaged over 10,000,000 timesteps for each realization and then further averaged across the five realizations. Figure S4 shows the variation of work of adhesion per unit area (w_{adh}) with surface/nanoparticle interaction strength (ε) used in the current study.

Effect of Drag

The current study simulates the vibration-induced detachment of a nanoparticle suspended in vacuum. In reality, vacuum conditions may not be possible. The Letter assumed that the nanoparticle dimensions are small enough to be comparable to the mean free path of gas/vapour at normal pressure and temperature. Therefore, to simplify the setup, the presence of gas molecules was omitted, and the simulations were performed in a vacuum. However, additional simulations were conducted to verify the effect of drag on nanoparticle detachment using the setup shown in Figure S5(a). This setup is similar to the standard configuration used in this study, but with argon atoms introduced into the domain to simulate the vapour. An additional barrier was placed at the top of the domain to prevent argon atoms from escaping through the periodic boundary and accumulating at the bottom of the vibrating surface. The number of vapour atoms was determined based on the density of argon at 20°C and 1 atm pressure. Additionally, a set of simulations was performed at 2 atm pressure to examine whether a higher gas density could affect particle detachment.

Figure S5(b) compares the optimal detachment cases in simulations with argon vapour to those conducted in a vacuum. The agreement between the results confirms that drag force has negligible significance at this scale. We also made order-of-magnitude estimates of the van der Waals energy and the energy loss due to drag. Figure S5(c) shows the variation of work of adhesion and drag energy as a function of nanoparticle size. The red markers in the

figure represent the work of adhesion of the particle used in this study, obtained from MD simulations. The red line in the figure is obtained by fitting $W_{adh} = c_1 \cdot l_c^2$ (c_1 is a constant) to the markers, as the work of adhesion is proportional to the contact area of the interface. The blue markers represent the work done by the drag force over a displacement of 1 nm of the particle, estimated as, $W_{drag} = s \cdot F_{drag}$. Here, s represents the displacement of the particle. The drag force, F_{drag} is calculated based on the characteristic velocity $\alpha\omega$ at the optimum detachment as

$$F_{drag} = \frac{1}{2} C_D \rho_{air} l_c^2 (\alpha\omega)^2, \tag{3}$$

where, $C_D = 1.05$ is the drag coefficient, $\rho_{air} = 1.204$ kg/m^3 is density of air. From the scaling law, for a given w_{adh} and amplitude of vibration, $l_c(\alpha\omega)^2 =$ constant. Therefore, Equation 3 indicates that the work done by the drag force varies linearly with l_c. To extend the comparison between drag energy and work of adhesion to larger particle sizes, the blue line is obtained by fitting $W_{drag} = c_2 l_c$ (c_2 is a constant) to the data points calculated for the particle sizes considered in the current study. S5(c) clearly shows that the energy loss due to drag is multiple orders of magnitude lower than the van der Waals energy at the lengthscales considered in the Letter.

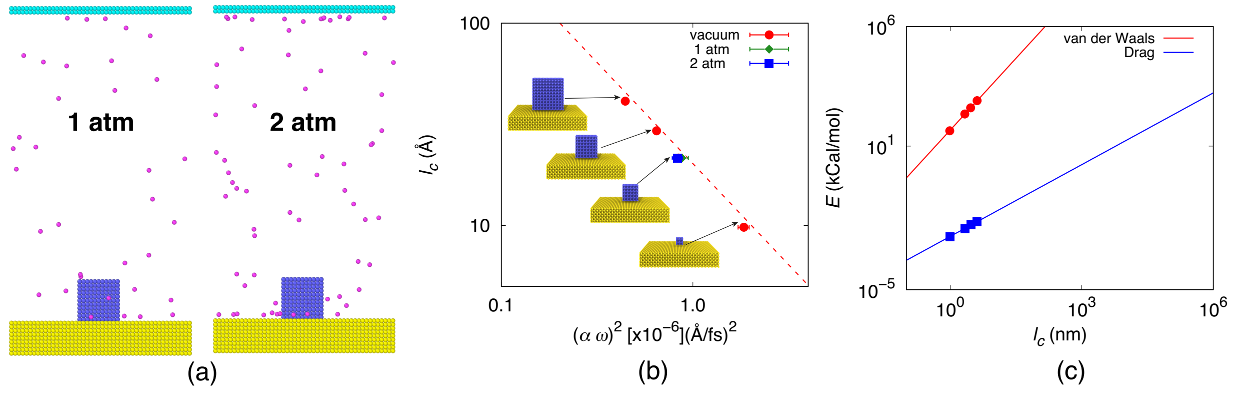

Figure S5: (a) Initial configuration of the domain for pressures of 1 atm and 2 atm. (b) Comparison of scaling for pressurised domain with original vacuum domain, showing negligible differences. (c) energy estimates of van der Waals and drag forces for varying nanoparticle size, showing that van der Waals forces are dominant for these systems.

Effect of Electrostatic Forces

All the MD simulations in the paper only considered LJ interactions between the surface and the nanoparticle. The scaling law developed in the paper was then validated using MD simulations, reproduced below:

$$l_c(\alpha\omega)^2 = \frac{(w_{\mathrm{adh}})_{\mathrm{LJ}}}{\rho\left(\frac{1}{2} + \frac{\alpha'}{\alpha}\right)}, \tag{4}$$

where ρ is the particle density, w_{adh} is the area-normalised work of adhesion, l_c is the characteristic length obtained by dividing the volume by the cross-sectional area, $l_c = V/A$, and α'/α represents the normalized displacement of the surface in its oscillatory motion.

To ensure that the proposed scaling is indeed universal, an additional set of simulations was conducted where electrostatic interactions were introduced along with van der Waals interactions (i.e., Lennard-Jones potential). The electrostatic force is modeled using the Coulombic pairwise interaction, given as

$$E_{elect} = \frac{C \cdot q_i q_j}{\epsilon_0 r_{ij}}, \tag{5}$$

where, C is an energy-conversion constant, q_i and q_j are the charges on the two interacting atoms, ϵ_0 is the dielectric constant of vacuum, and r_{ij} is the distance between the two charges. Negative charges are artificially assigned to the atoms of particle, while the atoms of surface are assigned positive charges. The charges are evenly distributed within the particle as well as on the surface atoms. It is also ensured that the net charge of the simulation domain is zero. This simulation setup therefore incorporated a combination of short-range interactions that decay quickly with distance from surface, and longer range electrostatic interactions that decay more gradually with distance. The LJ interaction strength between the particle and the surface was fixed at $\varepsilon = 0.3$ kCal/mol, and the charges on the atoms of the particle and the surface were adjusted in such a way that the total work of adhesion was equivalent to a case where the total interaction strength between the surface and the particle corresponded

to $\varepsilon = 0.5$ kCal/mol (the additional 0.2 kCal/mol, or 40 % of the total interaction strength was thus electrostatic in origin). Simulations were performed by keeping the amplitude fixed at 5 Å, while the frequency of vibration was varied to obtain the optimum lift-off. The cut-off for the electrostatic interactions were kept same as the LJ interactions (15 Å) for simulations with different particle sizes.

It is observed that the dynamics of particle dislodging remain the same as original cases in the Letter. The only difference is that, due to the gradually changing strength of the electrostatic interactions as the particle moves away from the surface, the surface performs some additional work while moving downward from its highest position (in the case of LJ interactions, negligible work is performed as the surface moves downward). This reduces the energy requirement for particle detachment, and can be calculated based on the formalism shown in Equation 4 of the Letter. As the surface undergoes sinusoidal vibrational motion while the particle is assumed to be fixed, it accelerates while moving down with a magnitude of $|\omega^2 y|$ at a displacement y. The magnitude of the force on the particle at y, due to the motion of the surface, is $|m\omega^2 y|$. Considering the displacement of the surface from the end of the upward motion ($y = \alpha$) to the mean position of vibration ($y = 0$), the work done can be expressed as

$$W_S = \int F(y)\mathrm{d}y = \int_{y=\alpha}^{y=0} m\omega^2 y \mathrm{d}y = \left|\frac{1}{2}m\omega^2\alpha^2\right|. \tag{6}$$

The above work can be considered as the energy gained by the system and, therefore, included on the left-hand side of the energy balance shown in Equation 2 of the Letter, such that Equation 7 of the Letter becomes:

$$\frac{1}{2}m\omega^2\alpha^2 + \frac{1}{2}m(2\alpha\omega)^2 = \frac{1}{2}m(\alpha\omega)^2 + \frac{1}{2}m\omega^2\left(\alpha^2 - \alpha'^2\right) + W_{\mathrm{adh}} + \frac{1}{2}m\omega^2(\alpha - \alpha')^2. \tag{7}$$

After simplification, the scaling for the combined van der Waals and electrostatic inter-

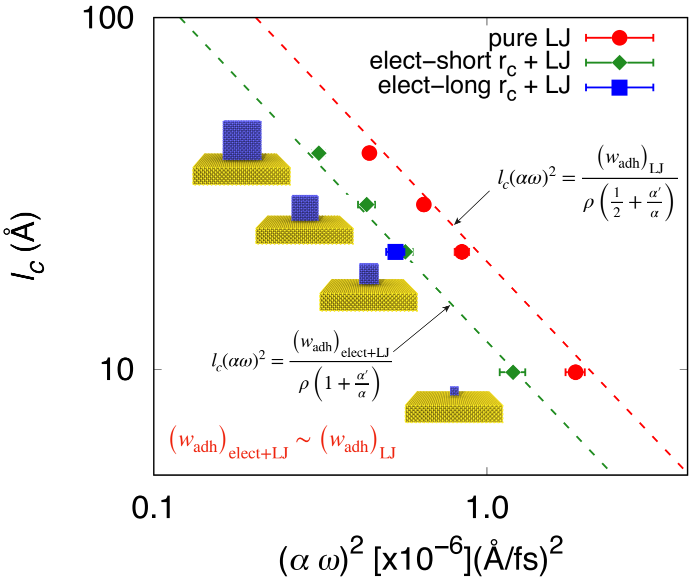

(a)

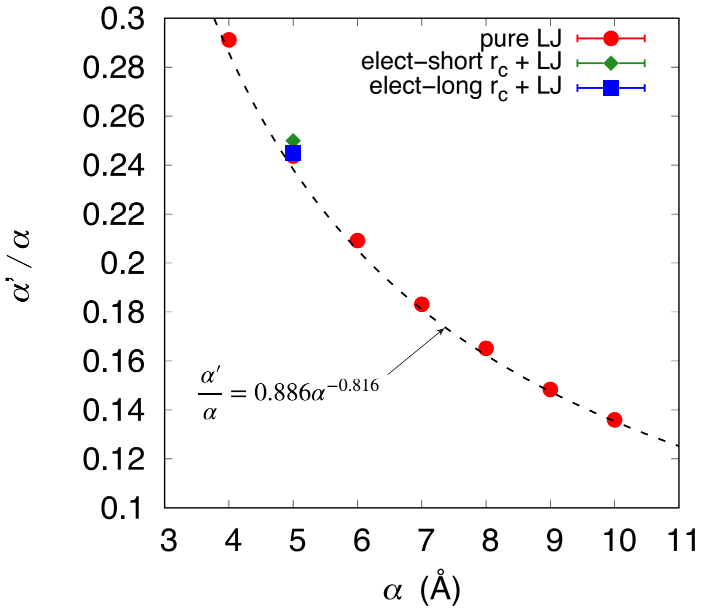

(b)

Figure S6: (a) Comparison of the derived scaling laws for both LJ-only (red dashed line) and combined LJ and electrostatic interactions (dashed green line) with MD simulation data for LJ-only (red circles), LJ and electrostatics with $r_c = 15$ Å (tilted green squares), and LJ and LJ and electrostatics with $r_c = 40$ Å (blue squares) are plotted. Embedded are images showing nanoparticle size. (b) Variation of the α'/α for optimum lift-off of the nanoparticle as a function of amplitude, α; the black dotted line represents the fitted curve over the averaged α'/α from all interaction strengths.

actions can be given as

$$l_c(\alpha\omega)^2 = \frac{(w_{\mathrm{adh}})_{\mathrm{elect+LJ}}}{\rho\left(1 + \frac{\alpha'}{\alpha}\right)}, \tag{8}$$

Comparing the scaling equation from the paper assuming only LJ interactions (Equation 4 above) and the scaling with electrostatics included (Equation 8) is instructive. The only difference is that the denominator on the right-hand side changes from $\rho\left(\frac{1}{2} + \frac{\alpha'}{\alpha}\right)$ to $\rho\left(1 + \frac{\alpha'}{\alpha}\right)$ to account for the reduced energy requirement of the particle at the end of the upward motion of the surface. Figure S6(a) shows the comparison between the scaling for pure van der Waals interactions and the combined electrostatic and van der Waals interactions. The dotted lines represent Equations 4 and 8, whereas the markers represent the results from the MD simulations. The close agreement between the MD results and Equations 4 and 8 validates our theory. It can be further observed from Figure S6(b) that the non-dimensional displacement, α'/α, remains unaltered regardless of the type of interactions, further reinforcing the universality of the scaling law.

A separate set of simulations with a much larger electrostatic cutoff of $r_c = 40$ Å were run to investigate the effect of cutoff on our results. Note that the simulations with the larger r_c were computationally very expensive. Therefore, the initial equilibration time was reduced from 10 ns to 5 ns, and the five realizations used to obtain the statistics were separated by 1 ns instead of 2 ns used for all the other simulations in this study. However, sufficient checks have been performed to ensure that this does not affect the simulation results. Note that the S6(b) shows that choice of electrostatic r_c does not influence the results.

References

(1) Plimpton, S. Fast parallel algorithms for short-range molecular dynamics. *Journal of computational physics* **1995**, *117*, 1–19.

(2) Hoover, W. G. Canonical dynamics: Equilibrium phase-space distributions. *Physical review A* **1985**, *31*, 1695.

(3) Martyna, G. J.; Klein, M. L.; Tuckerman, M. Nosé–Hoover chains: The canonical ensemble via continuous dynamics. *The Journal of chemical physics* **1992**, *97*, 2635–2643.

(4) Martyna, G. J.; Tuckerman, M. E.; Tobias, D. J.; Klein, M. L. Explicit reversible integrators for extended systems dynamics. *Molecular Physics* **1996**, *87*, 1117–1157.

(5) Kamberaj, H.; Low, R. J.; Neal, M. P. Time reversible and symplectic integrators for molecular dynamics simulations of rigid molecules. *The Journal of Chemical Physics* **2005**, *122*, 224114.

(6) Miller Iii, T.; Eleftheriou, M.; Pattnaik, P.; Ndirango, A.; Newns, D.; Martyna, G. Symplectic quaternion scheme for biophysical molecular dynamics. *The Journal of chemical physics* **2002**, *116*, 8649–8659.
